# Changes in viral load and HBsAg and HBeAg status with age in HBV chronic carriers in The Gambia

**DOI:** 10.1186/1743-422X-5-49

**Published:** 2008-04-16

**Authors:** Maimuna E Mendy, Samuel J McConkey, Marianne AB Sande van der, Sarah Crozier, Steve Kaye, David Jeffries, Andrew J Hall, Hilton C Whittle

**Affiliations:** 1 Medical Research Council Laboratories, Atlantic Boulevard, PO Box 273, Banjul, The Gambia; 2 Royal College of Surgeons in Ireland, 123 St. Stephen's Green, Dublin 2, Ireland; 3 National Institute for Public Health and the Environment, Bilthoven, The Netherlands; 4 Medical Research Council Epidemiology Resource Centre, University of Southampton, Southampton, UK; 5 Imperial College, London, UK; 6 London School of Hygiene and Tropical Medicine, Keppel Street, London, UK

## Abstract

**Background:**

Little is known about changes in hepatitis B viral load (HBV DNA) in relation to age in Africa. The aim of this study is to determine the natural course of HBV chronic infection, particularly in relation to sequential changes in serum HBV DNA levels and hepatitis B surface (HBsAg) antigen/hepatitis e antigen (HBeAg) status by age.

**Methods:**

The study was conducted on 190 HBV chronic carriers, aged 1–19 years who were followed for 19 years. 160, 99 and 123 were traced at 5, 9 and 19 years later. All available samples were tested for HBsAg and HBeAg, whilst 170, 61, 63 and 81 were tested for HBV DNA at the baseline, and at 5, 9 and 19 years following recruitment.

**Results:**

In general HBeAg which correlated with high levels of HBV DNA was lost at a much faster rate than HBsAg. 86% of the carriers who were recruited at the age of 1–4 yrs lost HBeAg by the age of 19 years compared to 30% who lost HBsAg. HBeAg negative carriers had serum HBV DNA levels of < 10^5 ^copies per mL, HBV DNA positivity declined from 100% in 1–4 yrs old carriers at recruitment to 62.5%,60% and 88% at 5, 9 and 19 years respectively following recruitment.

**Conclusion:**

After 19 years of follow up, the majority of HBV surface antigen carriers had lost HBeAg positivity and had low levels of viral replication. However small proportions (10–20%) retained HBeAg and continue to have high levels of viral replication.

## Introduction

Hepatitis B virus (HBV) is the leading cause of viral hepatitis in humans worldwide. Currently over two billion people have evidence of previous HBV infection and 350 million have become chronic carriers of the virus, 60 million of them residing in Africa [1]. There seem to be differing risks for HCC by geographical location, with higher risk recorded in countries in sub Saharan Africa and Asia compared to Europe [2-4]. Chronic HBV infection is a major cause of liver disease and is strongly associated with the development of hepatocellular carcinoma (HCC) [4]. Other risk factors for HCC include, male gender, co-infection with hepatitis C virus (HCV) [5], alcohol abuse [6], aflatoxin exposure [7,8] and HBV genotype [9].

Approximately one third of HBV carriers will progress to cirrhosis and 25% will develop HCC [10]. The risk of HCC is six times higher in patients who are persistently HBeAg positive than in HBeAg negative patients [11] and twelve times higher in patients with high DNA viral load (> 10^6 ^copies per mL)[12]. Most of the studies of the association of HBeAg and viral load with chronic liver diseases and HCC have been conducted in Southeast Asia. There is no comparable data in Sub Saharan Africa.

HBV infection is endemic in The Gambia with 15–20% unvaccinated Gambians chronically infected [13]. Liver cancer remains the commonest cause of death in adult males in The Gambia [14]. Studies of HBV infection conducted in two neighbouring villages of Keneba and Manduar (situated in West Kiang district) in the period before 1986–1990, when the national vaccination programme in The Gambia commenced, have shown that infection was uncommon in infants under the age of 6 months and sibling-to-sibling transmission rather than perinatal or vertical transmission was of major importance [13,15].

Other studies have shown that early in the carrier state, HBV infected individuals test positive for hepatitis B surface antigen (HBsAg) and hepatitis B e antigen (HBeAg) and have high levels of HBV DNA in their serum [16,17]. However the serum of older carriers, have detectable antibody to HBeAg (anti-HBe), which appears following clearance of HBeAg and is associated with low levels of HBV DNA [18,19].

The natural history of HBV infection is complex and variable and is greatly influenced by the age at infection and the level of HBV replication. The diversity of clinical outcome of HBV, either resolution (acute infection) or persistence (chronic carriage) of infection is dependent on the host immune response to the virus [20].

Important changes are occurring which are impacting on the natural history of HBV infection. These changes include vaccination programmes [21,22] and the development of mutant forms of the virus mainly pre core (pre-C) and basal core promoter (BCP) mutants [23].

The aim of the present study is to determine the natural history of chronic hepatitis B infection, particularly the changes in viral load, in The Gambia, where horizontal transmission occurs in childhood. In this manuscript we report HBV DNA levels and HBsAg and HBeAg status in chronic carriers who were identified at various ages and followed for a period of 19 years.

## Materials and methods

### Study population

Between 1972 and 1974 and in 1984, sero-surveys of HBV infections were conducted in inhabitants of the two villages of Keneba and Manduar and the pattern of childhood hepatitis was described [24-26]. The two villages are situated 8 kilometres apart and the inhabitants are of similar ethnic and religious background. Accurate demographic data have been kept since 1951, date of births have been recorded for every individual born after 1951.

During the 1984 survey 82 chronic carriers were identified in Keneba and 108 in Manduar. They have been followed at 3 time points 5 (in 1989), 9 (in 1993)and 19 (in 2003) years after recruitment [21,22,26]. The number and age distribution of the carriers at baseline was 68 age 1–4 years, 66 age 5–9, 38 age 10–14 and 18 age 15–19 years.

Blood samples collected at baseline in 1984 were tested for HBV marker (HBsAg, HBeAg if HBsAg is positive and anti HB core antibody), levels of HBV DNA and liver function tests (AST and ALT). During the follow up surveys blood samples were retested for HBV markers and HBV DNA levels. Liver enzymes were measured at baseline only.

Because the study is in the format of a cross sectional survey we were unable to establish the actual time of initial infection of the older children. However previous studies suggest that the majority were infected before the age of 10 years [13,15].

### HBV serology

Samples collected in 1984, 1989 and 1993 were tested for HBsAg by reverse passive haemaglutination assay (RPHA) (MUREX, Dartfort, UK) and by radioimmunoassay (RIA) (DiaSorin Biomedica, Sallugia, Italy). In 2003 following the discontinuation of RPHA and RIA assays, the samples were tested for HBsAg by Determine™ (Abbott laboratories), a visually read, qualitative immunochromatography assay. There was good correlation between the determine™ test and the RPHA and RIA assays. Samples positive for HBsAg were tested for HBeAg by RIA between 1984 and 1993 and later by enzyme immunoassay (EIA) (DiaSorin, Biomedica, Sallugia, Italy) after the discontinuation of RIA assay by DiaSorin. There was good correlation between the EIA method and the RIA assay.

### HBV DNA quantification by real time PCR

HBV DNA was measured by quantitative real-time PCR according to a previously described method [16]. Blood samples were collected and transferred into plain tubes and serum was separated and stored immediately at -20°C (for sample collected in 1984 and 1989) or -70°C (for samples collected in 1993 and 2003) until needed. DNA was extracted from stored sera which have been kept frozen for a period of 1 to 20 years. The assay was carried out using commercial SYBR-Green reaction mix (Qiagen, Hilden, Germany) and primers specific to the S gene designed to amplify a 98 base pair product. Thermal cycling was performed in an ABi 5700 sequence detection system (PE Applied Biosystems, Warrington, UK). The detection limit of the qPCR assay was 40 copies per mL and quantified accurately samples with greater than 2.6 × 10^2 ^DNA copies per mL. Test samples falling above the top of the standard curve were re-assayed at a dilution of 1:100. The assay was 100% specific when tested against HBV seronegative sera from ten subjects and coefficient of variation obtained from intra-and inter assay was 1.08 and 1.72 respectively. We considered serum HBV DNA levels greater than 10^5 ^copies/mL as high viral load and less than 10^5 ^copies as low viral load.

### Measurement of liver enzymes

Sera from blood samples were tested for ALT and AST aminotransferases by use of a Roche Cobas Mira Autoanalyser.

### Data management and statistics

Demographic data, subject's identification, date of birth, serology and results from biochemical tests were imported from the various databases developed during previously longitudinal studies, into an excel database created for the present study. The data for HBV DNA results were exported as an Excel spreadsheet into an Access database from the ABi real time machine after the PCR amplification and quantification.

Linear trend was tested by fitting a generalized estimating equation to allow for multiple responses within subjects using a logistic link with interchangeable correlation structure. For those analysis with trends having significant curvature, the rate difference between the first two time points was tested. Log rank tests were used to test the significance of relationships found. Analysis was performed using Stata 8.0.

## Results

### Prevalence of HBeAg and changes in viral load in HBsAg chronic carriers aged 1–4 years

Sixty-eight HBsAg carriers aged 1–4 yrs were identified in 1984. Forty-six (67.6%) of the 68 carriers were males and 22 were females, 10 (16.2%) had raised AST (> 44 mIU/mL) and 4 (5.9%) had raised ALT (> 44 mIU/mL) at recruitment. In 3 carriers both indices were raised.

Table 1a shows changes in HBV serological markers and HBV DNA detection in the 68 children identified at age 1–4 yrs. The proportion positive for HBsAg and HBV DNA fell sharply over the first five years then stabilized there after. In contrast, HBeAg prevalence dropped continually over the years (p vaue for trend < 0.001).

**Table 1:** a. Changes in HBsAg, HBeAg and HBV DNA prevalence with time in carriers detected between 1–4 years of age

	Years after recruitment			
Serological markers	0	5	9	19

HBsAg sero positivity (%)	68/68* (100)	42/60 (70.0%)	33/47 (70.2%)	37/53 (70.0%)
HBeAg seropositivity (%)	57/68* (83.8%)	25/42 (59.5%)	12/33 (36.3%)	5/53 (13.6%)
HBV DNA positive	62/62* (100%)	15/24 (62.5%)	20/39 (60.6%)	30/34 (88.2%)

**b)**				
**HBeAg positive carriers**				

	0	5	9	19

Those with detectable HBV DNA	46/46 (100%)	9/9 (100%)	6/7 (85.7%)	5/5 (100%)
Those with viral load > 10^5 ^copies/mL)	46/46 (100%)	9/9 (100%)	6/7 (85.7%)	5/5 (100%)
Geometric Mean HBV DNA (copies/mL)	4.5 × 10^8^	1.1 × 10^8^	4.4 × 10^8^	8.4 × 10^8^
Median HBV DNA (copies/mL)	1.0×10^9^	3.5×10^8^	1.5×10^9^	5.0×10^8^
IQR (copies/mL)	5.0 × 10^8^–2.0 × 0^9^	5.0 × 10^7^–6.5 × 10^8^	5.5 × 10^8^–6.5 × 10^9^	4.0 × 10^8^–2.5 × 10^9^

**c)**				
**HBeAg negative carriers**				

	0	5	9	19

Those with detectable HBV DNA	11/11 (100%)	2/7 (28.6%)	10/14 (71.4%)	28/31 (90.3%)
Those with high viral load (> 10^5 ^DNA copies/mL)	2/11 (18.2%)	1/7 (14.2%)	1/14 (7.1%)	3/31 (9.6%)
Geometric mean HBV DNA (copies/mL)	7.7 × 10^7^	2.1 × 10^8^	1.8 × 10^3^	4.2 × 10^3^
Median HBV DNA IQR (copies/mL)	2.0 × 10^4^	2.0 × 10^5^	2.0 × 10^3^	1.0 × 10^3^
IQR (copies/mL)	5.0 × 10^3^–8.0 × 10^4^	**3.6 × 10^4^–5.2 × 10^8^	1.0 × 10^3^–1.0 × 10^4^	4.0x × 10^2^-7.5 × 10^3^

*The p-values for difference in prevalence between the first two time points are < 0.006 for HBsAg, < 0.001 for HBeAg and HBV DNA respectively.**One carrier who is HBeAg negative had high serum DNA level (5.2 × 10^8^) at the second follow up point.

HBV DNA was detectable in the majority (85–100%) of HBeAg positive carriers and the majority of them had DNA levels greater than 10^5 ^copies/mL (Table 1b). Although HBV DNA was detected in 100% HBeAg negative carriers at baseline, the prevalence of HBVDNA positivity decreased at subsequent years following recruitment. DNA levels were mostly below 1.0 × 10^5 ^copies/mL in HBeAg negative carriers, only 7.1% to 18% of them have serum HBV DNA levels greater than 10^5 ^DNA copies/mL (Table 1c).

**Figure 1:**
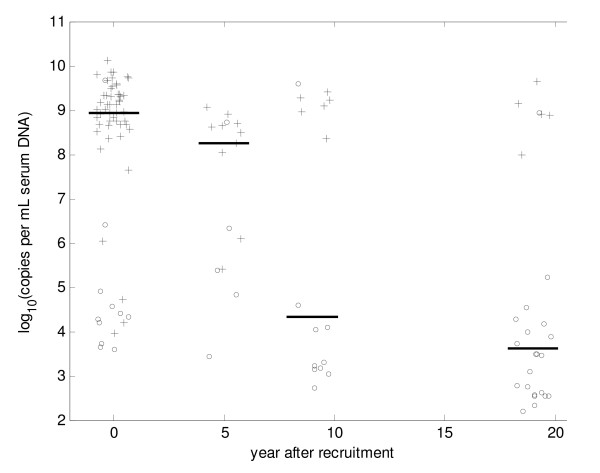
**Change in serum HBV DNA with time in HBsAg carriers detected between 1–4 years of age.** Horizontal bars represent median values. HBeAg positive carriers are represented by crosses and HBsAg negative carriers by the open circles.

The median viral load in the 68 younger carriers decreased significantly (p < 0.001) with time in this cohort (Figure 1). There was 1.0 log drop in viral load after 5 years and a further 4.0 log drop over the next 4 years thereafter levels stabilize. The difference in median viral load between carriers at age 5–9 yrs and at subsequent ages was highly significant (p < 0.001). Similar results were observed in the 10 carriers who had DNA test on each of the four time points (data not shown). However as the proportion of HBeAg positive carriers fall when we separate values for HBeAg positive carriers from HBeAg negative carriers we found no significant difference in serum HBV DNA levels between the different age groups.

High HBV DNA concentrations (> 10^5 ^copies/mL) were detected in only 3/31 (9.6%) HBeAg negative carriers compared to 5/5 (100%) HBeAg positive carriers aged 20–24 yrs (Tables 1b and 1c). Four of the HBeAg positive carriers had maintained high levels of HBV DNA over the period of 19 years.

### Prevalence of HBsAg and HBeAg and level of viral load with age in chronic carriers identified at older ages (5–19 yrs old)

Tables 2, 3 and 4 show data on the 122 older chronic carriers of all ages and recruited over the age of 5 years. Although this is a cross sectional study, it includes a longitudinal evaluation of carriers recruited between the ages of 1–19 yrs who were tested at 3 follow-up points. We were therefore able to compare longitudinal data using results from the younger carriers (aged 1–4 ys) with cross sectional data using results from the older carriers (5–19 yrs). The prevalence of HBsAg, HBeAg and HBV DNA positivity in the 122 older carriers followed similar patterns to that reported in the 1–4 years old cohort. Estimates of age specific seroconversion rates are shown in tables 5a–c. The comparison between the two cohorts revealed similar pattern of HBsAg and HBeAg seroconversion and serum HBV DNA clearance. Whilst the HBsAg seroconversion rates was high over the first 5 years in the each of the study groups there was little or no change in the subsequent periods following recruitment, HBeAg clearance rates were maintained at high levels in the different age groups. However the rate of serum HBVDNA clearance declined steadily with age.

**Table 2:** Changes in HBsAg prevalence with time in HBsAg positive carriers aged 5 years or older

	Years after recruitment			
Age at recruitment	0	5	9	19

5–9 (n = 66)	65/65 (100%)	49/62 (79%)	29/38 (76.3%)	38/43 (88.4%)
*10–14 (n = 38)	39/39 (100%)	21/28 (75%)	9/12 (75.0%)	16/20 (80%)
*15–19 (n = 18)	18/18 (100%)	6/10 (60%)	0/1	4/7 (57%)

*The p-values for the difference in prevalence between the first two time points for HBsAg is < 0.001

**Table 3:** Changes in HBeAg with age in HBsAg positive carriers aged 5 years or older

Subjects who remained HBeAg positive				
	Years after recruitment			

Age at recruitment	0	5	9	19

*5–9	38/65 (58.4%)	19/46 (41.3%)	10/29 (34.4%)	2/37 (5.4%)
*10–14	18/39 (46.0%)	9/21 (42.8%)	2/9 (22.2%)	0/15 (0%)
*15–19	6/18 (33.3%)	1/6 (16.6%)	-	0/4 (0%)

*The p-values for trend with time are 0.001, 0.001 and 0.376 for 5–9, 10–14 and 15–19 years old carriers respectively.

**Table 4:** Changes in median HBV DNA (copies/mL) in HBsAg positive carriers aged 5 years or over

DNA copies/mL at different time points				
	Years after recruitment			

Age at recruitment	0	5	9	19

5–9	3.4 × 10^8 ^(n = 60)	4.1 × 10^4 ^(n = 26)	*3.2 × 10^7 ^(n = 18)	1.70 × 10^3 ^(n = 32)
10–14	2.1 × 10^6 ^(n = 32)	9.0 × 10^7 ^(n = 9)	2.0 × 10^3 ^(n = 6)	2.8 × 10^3 ^(n = 11)
15–19)	6.9 × 10^5 ^(n = 16)	*2.5 × 10^8 ^(n = 2)	(n = 0)	3.4 × 10^3 ^(n = 4)

The (n) in brackets represent the number of subjects tested in the different age group category.

*The median values in this group is affected by

**Table 5:** The proportion of subjects who seroconverted or cleared serum HBV DNA by age of recruitment and interval after recruitment

a) HBsAg seroconversion			
Age (yrs) at recruitment	Interval after recruitment		
	
	First 5 years	5–9 years	10–19 years

*1–4	18/60 (30%)	1/33 (3.3%)	1/29 (3%)
*5–9	13/62 (21%)	0/27	0/22
10–14	7/23 (30%)	0/4	0/5
15–19	4/15 (27%)	0/0	0/0

b)			
HBeAg seroconversion			

Age (yrs) at recruitment	First 5 years	5–9 years	10–19 years
*1–4	12/36 (33%)	6/16 (37%)	7/10 (70%)
*5–9	10/29 (34%)	4/12 (33%)	5/7 (71%)
10–14	4/11 (36%)	1/2 (50%)	1/1 (100%)
15–19	1/4 (25%)	0/0	0/0

c)			
Serum HBV DNA clearance			

Age (yrs) at recruitment	First 5 years	5–9 years	10–19 years
*1–4	8/23 (34.7%)	4/12 (33.3%)	2/16 (12.5%)
*5–9	5/29 (17.2%	3/11 (27.2%)	1/12 (8.3%)
10–14	1/9 (11.1%)	0/2 (0%)	0/4 (0%)
15–19	1/3 (33.3%)	0/0	0/0

*The p-values for the test of trend over time for the first five years after recruitment is < 0.01, < 0.01 and < 0.05 for HBsAg, HBeAg and HBV DNA respectively.

We compared the HBeAg data with data from a cross sectional study conducted in Taiwan[27]. Similar patterns of rates of HBeAg seropositivity was obtained in young carriers (5–9 yrs) from the two countries, 64.3% reported in the Taiwanese study compared to 58.4% in our cross sectional study and 59.5% in the longitudinal study. However the rates of HBeAg positivity between the two cohorts are different in the older (> 10 years old) carriers. You et al., reported higher HBeAg prevalence of 50.8%, 26.7% and 23.3% in age groups 10–19 yrs, 29–29 yrs and 30–39 yrs respectively compared to 38.6%, 8.1% and < 5% in similar age groups of Gambian carriers.

## Discussion

Understanding the natural history of HBV is of public health importance since it will inform decision making in relation to the adoption of treatment strategies. Despite the vast amount of evidence on the role of HBV chronic carriage on the risk of development of HCC, there is little documented information on the natural history of HBV chronic carriage particularly in relation to viral load in people from sub Saharan Africa who have predominantly acquired HBV in early childhood, rather than during parturition or in adult life.

We showed that one third of young carriers clear HBsAg and progressed to 'immune clearance' phase in the first 10 years after infection; thus resulting to a short lived 'immune tolerance' phase. The remaining two thirds persistently test positive for HBsAg and continue to tolerate the virus. In contrast to HBsAg, clearance of HBeAg occurs at steady rates over the years. By the age of 24 years only 13.6% Gambian HBsAg carriers are positive for HBeAg. Our data supports previous cross-sectional studies, that the majority of chronically infected adult Gambians have undetectable HBeAg [17]. It was noted then and confirmed in the present study that HBeAg tend to wane with increasing age and the majority of adult carriers have maintained an inactive status. It is still not clear what factors are responsible for the loss of HBsAg or HBeAg which can occur spontaneously or following treatment with interferon or nucleoside analogues [28,29]. However, the risk for cirrhosis and HCC is low in inactive carriers with non-replicating HBV and these carriers have similar rates of liver-related morbidity and mortality when compared to uninfected individuals [3]. The lower rates of HBeAg seropositivity in older carriers in the present study compared to carriers from Taiwan relates to the reported differences in the epidemiology of HBV between sub-Saharan Africa and South east Asia. Perinatal transmission is the commonest route of transmission in Asia and affects young infants (< 6 months) whilst in Africa, horizontal or sibling to sibling transmission is the most important route of transmission and affects young children between 6–12 months of age [13,30].

Although our data suggests that HBV DNA levels decline with age among asymptomatic carriers it is beyond the scope of this study, because we did not have disease as an outcome, to determine whether low levels are maintained in people without liver disease or whether increased viral replication is restored in persons with liver disease. We were also unable to determine whether persistent high levels of HBV DNA are associated with liver disease.

Chen et al, were able to evaluate HBV DNA levels in a Taiwanese HBV cohort of asymptomatic carriers and HCC disease prior to HCC diagnosis [12]. They showed significant increased risk of HCC among those persons with levels > 10^5 ^copies/ml. They suggest that persistently elevated HBV DNA levels are the best predictor of HCC development. A prospective longitudinal study by Hui et al., showed that although a high serum HBV DNA may be associated with a lifelong higher chance of developing HCC or cirrhosis, carriers in the immune-tolerant phase who have high levels of HBV DNA, have low disease activity and low rate of disease progression. The rate of progression of fibrosis increased once the carriers progressed into the immune clearance phase [31].

It has been suggested by a liver cancer case control study conducted in The Gambia, that even a low level viremia (HBV DNA < 1.0 × 10^5 ^copies/ml) is significantly associated with HCC but risk for cirrhosis did not increase until serum HBV DNA levels reaches ≥ 1.0 × 10^5 ^copies/ml (Mendy et al., -in press). There is a small proportion of carriers in our study, who maintained high levels of HBV DNA (> 1.0 × 10^5^) they would have benefited from antiviral therapy. Although treatment is not recommended in carriers who are in the immune-tolerant phase, not only because of little benefit to the carriers but will encourage the development of viral resistance [20]. However such individuals would benefit from regular screening programmes and they should be monitored closely for progression to the immune clearance phase.

Replicative HBV infection is the stimulus for host immune responses leading to the chronic process of hepatocyte destruction and regeneration with development of fibrosis and eventually cirrhosis. As such, it may be that a certain threshold level of HBV replication is required to lead to development of cirrhosis. Serial sampling of HBV-infected persons with lengthy longitudinal follow-up, similar to the current study but with disease end points will allow investigators to fill in the gaps in our knowledge of the time sequence between HBV DNA levels measured remotely and proximately to development of cirrhosis or HCC.

HBV core mutations, particularly in the basal core promoter (BCP) region have been reported in asymptomatic carriers from The Gambia [23]. BCP mutants were implicated in the development of advanced liver diseases in the Gambia (Mendy et al.,-in press). Further studies are needed to determine the extent of the involvement of these mutants in development of HCC in The Gambia.

Apart from viral replication, HBeAg status and viral mutations, several other factors, including HBV genotypes, co infections with HIV or HCV and exposure to aflatoxin have been suggested to influence natural history of chronic carriage [4,32,33]. HIV and HCV infection are of low prevalence in The Gambia. (1.0% for HIV-1, 0.8% for HIV-2 and 2.0% of HCV) [32], Although HCV is present in only 2.0% of the Gambian population it contributes to 19.0% HCC cases mainly in the older age groups [4].

In conclusion, we charted the level of viral load and HBV serology marker in carriers over a period of 19 years. Our data confirms previous findings that viral load declined with age [17]. We further conclude that HBsAg clearance was not affected by the rate of reduction in concentration of viral load.

Despite immunotolerance, older HBV carriers clear HBeAg and partially control viral replication. Most Gambian chronic carriers are in the inactive HBsAg carrier state and once cleared; reactivation of HBeAg is uncommon.

## Authors' contributions

MM, SM, MVDS, AH and HW participated in the design of the study; HW and SM obtained funding support; MM and SK participated in the laboratory analysis; MM, SC and DJ Performed the data analysis; MM, HW, SM and MVDS and AH contributed to manuscript preparation. All authors read and approved the final manuscript.
